# Uncovering the microbiome landscape in sashimi delicacies

**DOI:** 10.1038/s41598-024-55938-1

**Published:** 2024-03-05

**Authors:** Cheng‑Wei Ho, Pei‑Ying Chen, Yi-Ting Liao, Yen-Fu Cheng, Han-Hsing Tsou, Tsung‑Yun Liu, Kung-Hao Liang

**Affiliations:** 1 Institute of Food Safety and Health Risk Assessment, National Yang-Ming Chiao-Tung University, Taipei, Taiwan; 2 Department of Medical Research, Taipei Veterans General Hospital, Taipei, Taiwan; 3 Institute of Biomedical Informatics, National Yang-Ming Chiao-Tung University, Taipei, Taiwan

**Keywords:** Food microbiota, Salmon, Metagenomics, Tilapia, Bacteria, Sequencing

## Abstract

It is widely believed that a significant portion of the gut microbiota, which play crucial roles in overall health and disease, originates from the food we consume. Sashimi is a type of popular raw seafood cuisine. Its microbiome, however, remained to be thoroughly explored. The objective of this study is to explore the microbiome composition in sashimi at the time when it is served and ready to be eaten. Specifically, our tasks include investigating the diversity and characteristics of microbial profiles in sashimi with respect to the fish types. We utilized the Sanger-sequencing based DNA barcoding technology for fish species authentication and next-generation sequencing for sashimi microbiome profiling. We investigated the microbiome profiles of amberjack, cobia, salmon, tuna and tilapia sashimi, which were all identified using the MT-CO1 DNA sequences regardless of their menu offering names. Chao1 and Shannon indexes, as well as Bray–Curtis dissimilarity index were used to evaluate the alpha and beta diversities of sashimi microbiome. We successfully validated our previous observation that tilapia sashimi has a significantly higher proportions of *Pseudomonas* compared to other fish sashimi, using independent samples (P = 0.0010). Salmon sashimi exhibited a notably higher Chao1 index in its microbiome in contrast to other fish species (P = 0.0031), indicating a richer and more diverse microbial ecosystem. Non-Metric Multidimensional Scaling (NMDS) based on Bray–Curtis dissimilarity index revealed distinct clusters of microbiome profiles with respect to fish types. Microbiome similarity was notably observed between amberjack and tuna, as well as cobia and salmon. The relationship of microbiome similarity can be depicted as a tree which resembles partly the phylogenetic tree of host species, emphasizing the close relationship between host evolution and microbial composition. Moreover, salmon exhibited a pronounced relative abundance of the *Photobacterium* genus, significantly surpassing tuna (P = 0.0079), observed consistently across various restaurant sources. In conclusion, microbiome composition of *Pseudomonas* is significantly higher in tilapia sashimi than in other fish sashimi. Salmon sashimi has the highest diversity of microbiome among all fish sashimi that we analyzed. The level of *Photobacterium* is significantly higher in salmon than in tuna across all the restaurants we surveyed. These findings provide critical insights into the intricate relationship between the host evolution and the microbial composition. These discoveries deepen our understanding of sashimi microbiota, facilitating our decision in selecting raw seafood.

## Introduction

Inhabitants in regions adjacent to the ocean benefit from a rich array of seafood offerings, which enables the development of many traditional seafood cuisines. Among them, sashimi, originated from Japanese culinary traditions, has gradually become one of the world’s most recognized and favorable type of seafood cuisine. Sashimi, a culinary delight, is a thinly and delicately sliced, bite-size fish meat often served with condiments such as soy sauce and wasabi. Some sashimi is flame-seared for adding flavor, while others are served raw for preserving its original taste. Sashimi can also appear in the form of Nigiri, which is sashimi served together with vinegar-flavored rice, forming a nice contrast in both color and taste, and ensuring visual and taste enjoyments. Sashimi has become increasingly popular among health-conscious consumers, as it is high in protein, low in cholesterol, and with nutritional components beneficial for the human^1^. Marine fish is rich in healthy omega-3 long-chain polyunsaturated fatty acids (n-3 LCPUFAs), and a wide array of highly bioavailable micronutrients such as vitamins A, B12, D and E, iodine, selenium, calcium, zinc and iron^2,3^. Benefits associated with the consumption of seafood include reduction of risks of cardiovascular disease^3^, increase of insulin sensitivity in diabetes patients^4^, anti-inflammatory effects^5^, and the lowering of blood pressure^6^.

Common types of fish used for sashimi include tuna, salmon, amberjack, and cobia. Tuna is a highly regarded and priced fish particularly in Japan. Bluefin tuna, yellowfin tuna and bigeye tuna are commonly used for sashimi, which usually appear in a deep red color and has a meaty flavor and a high fat content. Salmon sashimi, usually appeared as strips of bright orange color, has a light, mild flavor and a soft buttery texture. Salmon is a good source of omega-3 fatty acids. Amberjack sashimi, appear in white or pale pink color, is characterized by its firm, meaty texture and a mild, slightly sweet flavor. Cobia sashimi, which usually have a light pink or white color, has a slightly firm texture and a more pronounced, slightly sweet flavor. Due to the importance of seafood, we previously conducted a seafood substitution study in Taiwan, investigating identities of seafood products from accessible markets and restaurants using the DNA sequences in the MT-CO1 gene. We revealed a prevalent mislabeling problem with an average mislabel rate of 18.9%^7^ where tilapia was often used to substitute snapper, including snapper sashimi served in restaurants. Moreover, such falsely labeled sashimi were found to exhibit elevated *Pseudomonas* bacterial DNA levels, compared with other sashimi dishes^7^. This observation motivates us to conduct this extensive microbiome investigation of served sashimi dishes using independently acquired samples, not only to validate the previous finding but also to perform comprehensive profiling of microbiome of the served sashimi in Taiwan.

When we savor sashimi dishes, we not only enjoy the exquisite flavors and textures but also unwittingly partake in a diverse array of microorganisms into our bodies. The gut microbiota, a critical indicator of human health and plays a pivotal role in both well-being and disease, is supposedly influenced by the very food we consume^8^. As we appreciate the delectable slices of sashimi, we should also appreciate the role it might play in nurturing or altering our gut microbiome. Although fresh, high-quality sashimi dishes handled properly in a clean environment are generally considered safe to eat, consuming sashimi, being a raw food, can pose risks of foodborne diseases if pathogens like bacteria or eukaryotic parasites are present^9^. To prevent the risk of exposure to those pathogens, most of fish species used as ingredient for sashimi are saltwater fish, as the salt in the water creates a hypertonic solution, and the cold chain of marine fishery also prevents the growth of bacteria and survival of parasites^10^. This way, parasitic or symbiosis microbiota in the fish meat may have already been inactivated by the freezer soon after the fish were caught in the ocean. Nevertheless, a recent study in Lisbon, Portugal, evaluating the microbiological quality of take-away sushi found that 83.9% of samples were either unsatisfactory or borderline, with the presence of B. cereus and coagulase-positive Staphylococci at unsatisfactory levels^11^.

Apart from some anecdotal observations of people infected by Listeria, Vibrio and Salmonella when the food is not well prepared, the microbiota diversity in sashimi has not been comprehensively explored. Microbiome is a subject of great scientific interest with a wide diversity of application and different approaches of investigation. Ecological studies of microbiome often focus on a habitat with distinct physio-chemical properties^12^. Human microbiome studies address the comprehensive microorganisms for their role in health and disease^13^. Metagenomics is an approach of microbiome study, with a focus of microbial genetic DNA^14^. Widely used in environmental and biological studies, metagenomic techniques offer comprehensive insights into diverse microbial habitats^15,16^, including fish intestines and gills^17^. The metagenomics approach integrates molecular barcoding, next-generation sequencing, and big data analysis techniques, without using microbial culture procedures. Mitochondrial DNA of eukaryotic cells carries variants, particularly in regions of the Cytochrome C Oxidase 1 (MT-CO1) genes, which are frequently used as a reliable molecular barcode, enabling diverse biological classification and identification^18–20^. The 16S ribosomal RNA (16S rRNA) gene, on the other hand, are widely employed for in-depth microbiome analysis^17^. The total collection of microbes and their genomic elements as well as the biological behaviors are referred to as Microbiota^21^. The objectives of this study were to explore the microbiome composition in sashimi, employing DNA barcoding and microbiome profiling techniques. Specifically, our aims included investigating the diversity, the unique microbial profiles, and potential associations between fish species and their sashimi microbiomes. The landscape of sashimi microbiome will be illustrated using the 16 s rRNA amplicon sequencing method.

## Methods

### Sample collection/preparation

**Figure 1:**
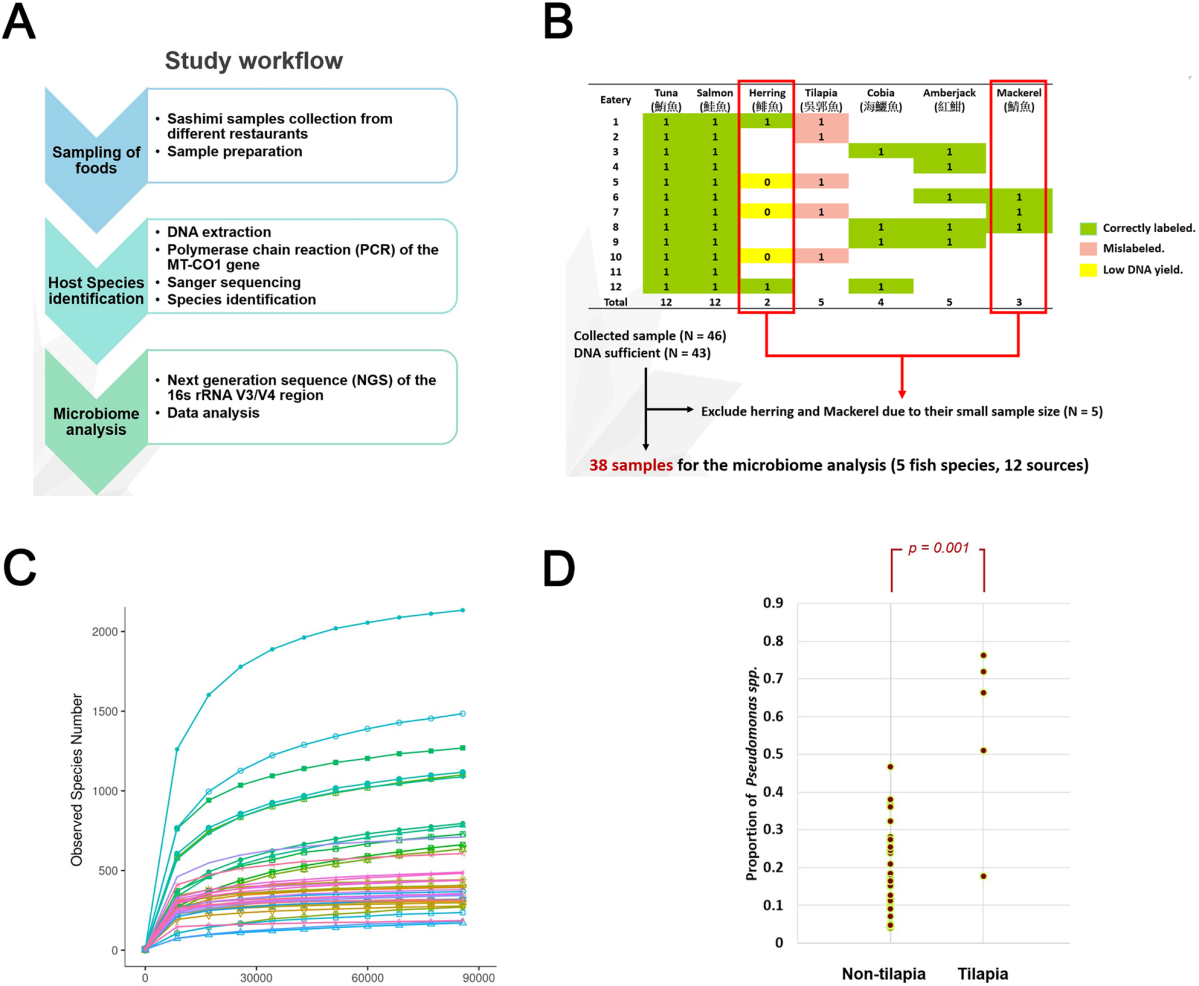
(**A**) Flowchart of the study, outlining the three major steps of this research including sashimi specimens collection; fish species identification using MT-CO1 sequences as barcodes; and microbiome profiling using 16S rRNA sequences. (**B**) The sashimi specimens were collected from 12 food providers/restaurants in Taiwan. After fish species identification, a total of 38 samples were selected for microbiome profiling (**C**) Rarefaction curves of all the investigated sashimi samples. The horizontal axis shows the sequencing depths. The vertical axis shows the number of identified operational taxonomic units (OTU). The saturation of the identified number of OTUs demonstrates that sequencing depth is adequate for a comprehensive analysis. (**D**) The relative abundance of *Pseudomonas* spp. in sashimi samples of tilapia are significantly higher than other fish types (Mann–Whitney test; P = 0.0010), validating our previous observations successfully.

This study aims to investigate the microbiota of sashimi samples at the timepoint when they were served to customers and ready to be eaten. The procedure of this study included sample collection/preparation, fish type identification and microbiome profiling (Fig. 1A). We collected a total of 46 new sashimi specimens of different fish types, including tuna, salmon, herring, tilapia, cobia, amberjack, and mackerel, from 12 local restaurants, sushi bars and food providers in Taiwan, between March and May 2020 (Fig. 1B). The sashimi specimens are acquired specifically to this study and have not been included in the previous study^7^. Among them, 3 samples have low DNA yields which prevented themselves from further investigations. The effective sample size of herring and mackerel ≤ 3 and thus were excluded. As a consequence, a collection of 38 samples with 5 different fish types from 12 collection sites were used for this microbiota analysis (Fig. 1B). In average, each fish type has 7.6 sample. Each site offers 3.1 samples. All these collected samples were documented through photographs.

Upon collection from vendors, the sashimi specimens were promptly transported to the laboratory and processed within two hours, minimizing the time after it is served. Also, during the transportation, the samples were stored in isothermal bags with ice packs within to help regulate and maintain a low temperature. We used separate bags for each sample to prevent any transfer of contaminants between different specimens. In instances where sushi comprised both sashimi and non-sashimi elements, like vinegar-flavored rice, we specifically gathered and analyzed solely the sashimi components for the purpose of this research. For example, when dealing with sushi, we collected samples of raw fish meat while excluding any accompanying vinegard rice. The specimens were cut into small pieces (5–10 g), ground and transferred into 1.5 mL microcentrifuge tubes for DNA extraction. DNA was extracted from 25 mg of ground tissue using the Qiagen QIAamp DNA Microbiome Kit (Qiagen, Hilden, Germany). The samples underwent lysozyme treatment and were subsequently subjected to 15 h of Protease K treatment. We performed regular sanitization of hands, work surfaces, and tools to minimize the risk of cross-contamination.

### Fish species identification

We use the same technical approach as in Chen et al.^7^ that all fish types are identified molecularly using the MT-CO1 DNA. Briefly, DNA-amplifying primers (Fish4F, Fish4R for most fish types; Fish3F, Fish5R for tuna) were designed for targeting conserved genomic regions, generating amplicons within the 520–625 nucleotide base range depending on the type of fish (Table 1)^7^. Subsequently, DNA extracted from fish samples underwent Polymerase Chain Reaction (PCR), involving denaturation, annealing, and extension steps (Table 1), followed by electrophoresis and DNA spectrometer analysis for quality control. Sanger sequencing, a classical DNA sequencing method that involves chain-terminating dideoxynucleotide incorporation during DNA synthesis, was then performed using both forward and reverse primers (Table 1). We did not use sample pooling approaches. Each sample was processed individually. The obtained sequences were then compared to references in NCBI GenBank and Barcode of Life Data Systems (BOLD) for molecular taxonomic identification^22,23^.

**Table 1:** Experiment settings, including DNA amplification primers and polymerase chain reaction (PCR) conditions used, for fish type identification.

	DNA amplification parameters for host species identification					
Sashimi labels	salmon, amberjack, cobia, snapper			tuna		
Forward	TATCTAGTATTTGGTGCCTGAGCCGG			CACGCCTTAAGCTTGCTCATCCGAGC		
Reverse	TCACCTCCTCCAGCAGGGTCAAAGAA			TCCCCTCCGCCTGCCGGGTCAAAGAA		
Stage	Condition			Condition		
Initial denaturation	95 °C	1 min		95 °C	1 min	
Denaturation	95 °C	30 s	35 cycles	95 °C	30 s	35 cycles
Primer annealing	60 °C	30 s		64 °C	30 s	
Primer extension	72 °C	40 s		72 °C	40 s	
Final extension	72 °C	5 min		72 °C	5 min	

### Microbiome investigation

High-throughput sequencing techniques enabled the comprehensive profiling of microbial communities present in each species. The extracted DNA was then used for microbiome profiling, using the DNA encoding the high-variable regions V3 and V4 of the 16S rRNA, a region widely used for metagenomics^24^. The DNA were amplified using primers combined with adapters (Table 2)^25,26^. The amplicon size was approximately 428 base pairs, with slight variations which can serve as barcodes for various microorganisms. Subsequently, we sequenced the DNA in the samples using the Illumina sequencing platform and MiSeq v3 sequencing chemistry, with 2.0 µg of DNA per sample. The read count for each sample is greater than 800,000. We did not use sample pooling approaches. Each sample is processed individually.

**Table 2:** The primers incorporating adaptors and the two sides of targeting sequences in the 16s gene for microbiome profiling using the next generation sequencing platform.

Primer	Adaptor	16s gene targeting sequence
Forward	5′-TCGTCGGCAGCGTCAGATGTGTATAAGAGACAG-3′	5′-CCTACGGGNGGCWGCAG-3′
Reverse	5′-GTCTCGTGGGCTCGGAGATGTGTATAAGAGACAG-3′	5′-GACTACHVGGGTATCTAATCC-3′

After sequencing, we employed FLASH software to merge the paired-end reads^27^. We used the UCHIME bioinformatics software to remove the portions of sequences containing adapters and eliminated chimeras generated during the processing steps^28^. The sequences then underwent quality filtering to generate effective tags (Supplementary Table 1). The effective tag sequences were then utilized for microbial taxonomy identification. Initially, we employed the UPARSE algorithm^29^ from USEARCH bioinformatics software (v 7.0) to cluster these effective tags with sequence similarity greater than 97% into Operational Taxonomic Units (OTUs)^30^. We then annotated the identified OTUs using the Ribosomal Database Project (RDP) classifier^31^, a Bayesian method that provides classification at the kingdom, phylum, class, order, family, and genus levels. This comprehensive annotation particularly at the genus level offers a detailed insight into the taxonomic composition within the sashimi microbiome. Furthermore, we aligned the sequences with microbial references from the NCBI GenBank to obtain more detailed annotations. The relative abundance of identified microbial OTUs from the DNA sequences of sashimi samples are shown in Supplementary Table 2.

## Results

### A higher proportion of *Pseudomonas* spp. DNA exist in tilapia compared to other fish

Fish types in this microbiome study include amberjack (*Seriola dumerili*, *Seriola quinqueradiata*), cobia (*Rachycentron canadum*), salmon (*Salmo salar*), tuna (*Thunnus* sp.) and tilapia (*Oreochromis niloticus*) which were all identified using the MT-CO1 DNA sequences regardless of their product names on the menu. Salmon and tuna, two popular type of sashimi, were available from all the 12 eateries where we collected the samples, while other fish types were only available in some restaurants (Fig. 1B). Like the observation of the previous study^7^, all the tilapia samples that we acquired from the eateries were fraudulently labeled as snapper without exceptions.

OTUs, identified by the 16 s rRNA DNA sequence reads, are the basic units of our analysis. Rarefaction curves were generated using the number of identified OTUs in random subsets of sequences, each representing varying proportions from 10%-100% (Fig. 1C). The number of identified OTUs are saturated when the sequencing depth reached ~ 90,000 (Fig. 1C). Hence, the final sequencing depth (> 90,000) is adequate for supporting a fair analysis. At this sequencing depth, a total of 5377 OTUs (units of microbiome generated in the analysis process) were identified (Supplementary Table 2), with 3662 OTUs in the salmon samples, 1871 OTUs in the tuna samples, 957 OTUs in the amberjack samples, 813 OTUs in the cobia samples, and 650 OTUs in the tilapia samples. The OTUs were then annotated to facilitate subsequent analysis. Using the newly acquired samples this time, we showed again that the proportion of *Pseudomonas* spp. is significantly higher in tilapia than in other fish types, confirming our previous observation (Mann–Whitney test; P = 0.0010; Fig. 1D).

### Fish types are an important factor affecting microbiome in sashimi

**Figure 2:**
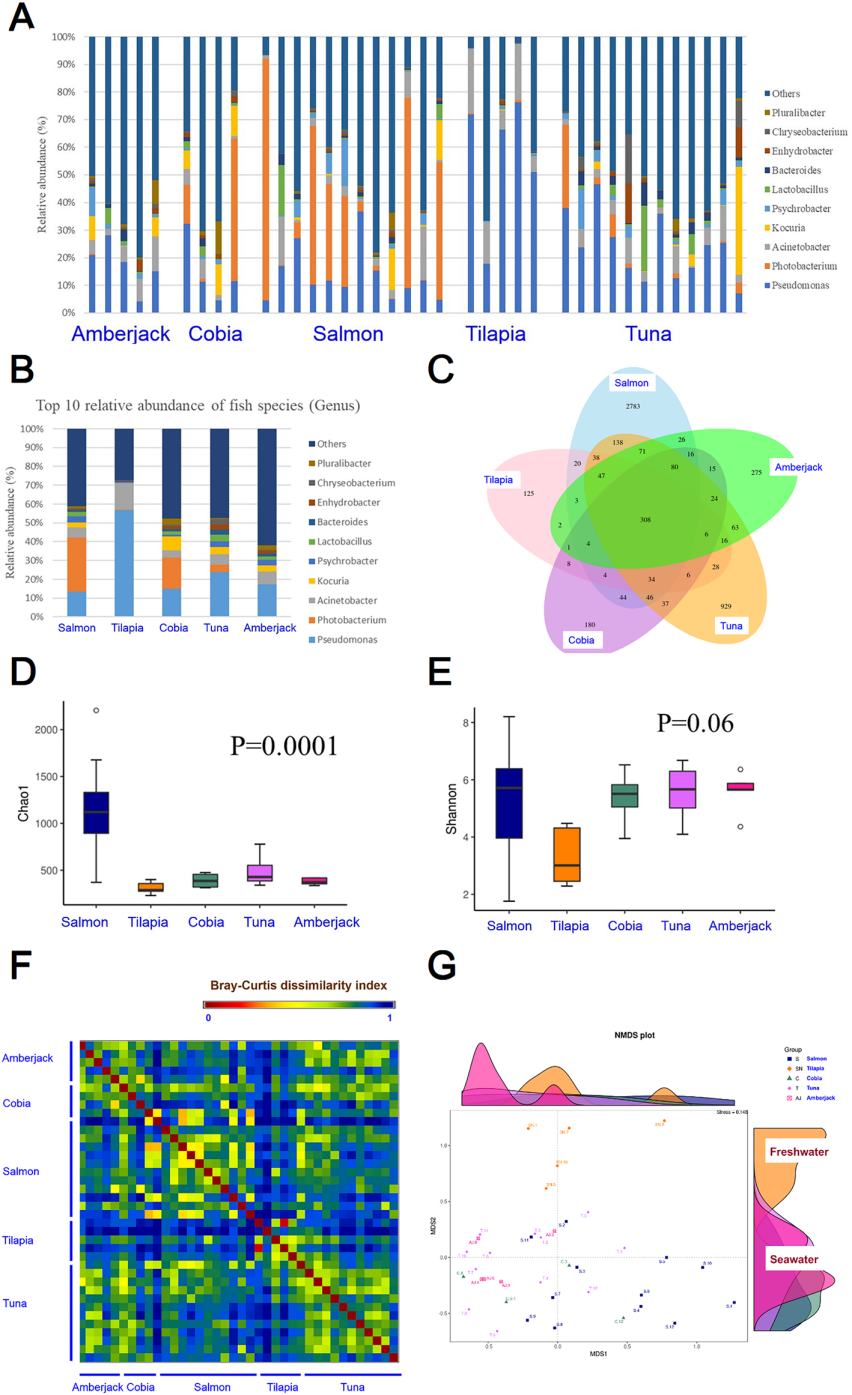
(**A**) The distribution of 10 leading microbiome at the genus level with the highest average relative abundance across all the collected sashimi samples. (**B**) Relative abundance of microbiome of five fish types. (**C**) Venn diagram displaying shared OTUs among the five fish types. (**D**) Alpha diversity analysis using the Chao1 index, indicating the richness of microbiota in different fish types. Salmon has the highest diversity of microbiome in the fish types that we analyzed. (**E**) Alpha diversity analysis using the Shannon index. Salmon, cobia, tuna, and amberjack exhibit similar Shannon diversity values, while tilapia's microbiome richness is comparatively lower. (**F**) Comparison of microbiome using Bray–Curtis dissimilarity index of all sample pairs and illustrated as a heatmap. The average value and range of the index within each fish type are: Amberjack 0.76 (0.67–0.89); Cobia 0.76 (0.64–0.84); Salmon 0.72(0.35–0.92); Tilapia 0.66 (0.09–0.93); Tuna 0.74 (0.55–0.90). (**G**) The Non-Metric Multidimensional Scaling plot, based on Bray–Curtis dissimilarity index, specifically illustrated several distinct clusters of microbiome corresponding to different fish types, especially between seawater and freshwater fishes residing in two different aquatic environments.

We aim to elucidate microbiomes to uncover patterns in microbial diversity and abundance. The newly acquired samples allowed us to perform a comprehensive analysis for contributing factors of sashimi microbiome. A total of 1232 OTUs were annotated successfully to the genus level (Supplementary Table 3). We started from showing the microbiome composition using the top 10 genus with the highest relative abundance across all sashimi samples (Fig. 2A). The less abundant microbes are aggregately presented together in the “other” category. The microbiome composition of different fish types are shown in Fig. 2B, showing that fish types significantly influence the microbiome composition in sashimi. The number of shared and dish type-specific microbiome are also presented as a Vann diagram (Fig. 2C). We further use the diversity matrices to show the microbiome diversity within or between the sashimi samples, and referred to as the alpha diversity and the beta diversity respectively. The microbiome within samples (i.e. alpha diversity) was analyzed using the Chao1 and Shannon indexes. The Chao1 index, a measure of the richness of microbiome in each sample, manifested distinct distributions with respect to fish types (Fig. 2D; Kruskal–Wallis test, P = 0.0001). Among the five fish types evaluated, salmon has the highest richness of microbiome (Fig. 2D). The Shannon diversity index, on the other hand, gives more weights to rare species. Shannon diversity index of tilapia’s microbiome is lower than those of other fishes, including salmon, cobia, tuna and amberjack which all have similar Shannon diversity index values (Fig. 2E).

We also calculated pairwise Bray–Curtis dissimilarity index, assessing beta diversity and dissimilarity of pairs of samples which may belong to the same or different fish types. The pairwise values are represented as a heatmap (Fig. 2F). Microbiome of the same fish types are similar to each other, represented as bright yellow color in the heatmap (Fig. 2F). The averages and ranges of the Bray–Curtis dissimilarity index of these fish types are: Amberjack 0.76 (0.67–0.89); Cobia 0.76 (0.64–0.84); Salmon 0.72(0.35–0.92); Tilapia 0.66 (0.09–0.93); Tuna 0.74 (0.55–0.90). No statistically significant difference was found between the distributions of Bray–Curtis dissimilarity index of the five different fish types. The Non-Metric Multidimensional Scaling (NMDS) plot, constructed by the Bray–Curtis dissimilarity index, illustrate several distinct clusters of different fish species. Fish inhabited in freshwater (tilapia) and seawater are separated clearly in the plot (Fig. 2G).

### The landscape of sashimi microbiome with respect to fish types

**Figure 3:**
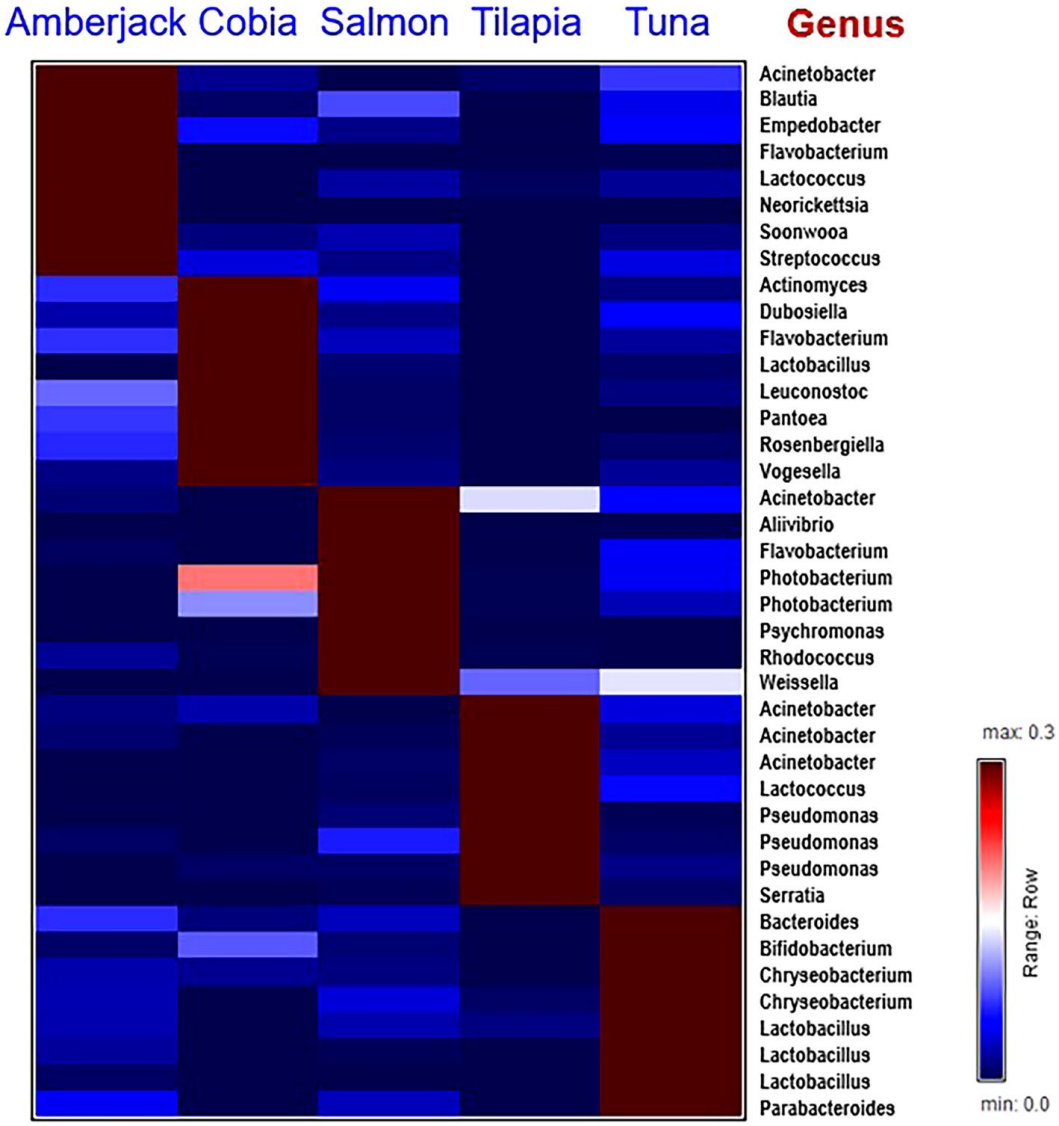
Microbiome landscape of sashimi, represented by the top 8 genera with the highest relative abundance detected in amberjack, cobia, salmon, tilapia and tuna in contrast to other fish types, respectively. The color indicates the average relative abundance of a microbial in a type of fish.

We then presented the microbiome landscape using the dominant genera that can characterize the sashimi fish types. We collected the microbial genera with the top 8 highest relative abundance in amberjack, cobia, salmon, tilapia and tuna, in contrast to the other fish types, for visualizing the microbiome compositions representing the fish types (Fig. 3; Supplementary Table 4). These genera are referred to as the fish-type characteristic genera. Amberjack harbors a microbial community rich in marine-associated genera like Flavobacterium, Acinetobacter, and Soonwooa, as well as the probiotic Lactococcus and Streptococcus. Pathogens such as Acinetobacter^32^, Empedobacter^33^, and Neorickettsia^34^ poses a potential health concern. Cobia, a denizen of marine realms, exhibits a distinctive microbiome featuring Flavobacterium and Vogesella, as well as Lactobacillus and Leuconostoc which are probiotics. While this microbime suggest a pretty low pathogenic potential, conditions may render Pantoea as opportunistic pathogens^35^. Salmon, thriving in marine and freshwater environments at different phases of their lifetimes, showcases a microbiome with Aliivibrio^36^, Flavobacterium^37^, Photobacterium, and Psychromonas. Aliivibrio, while not typically human pathogens, can pose risks to marine organisms^36^. Aliivibrio salmonicida is identified as the etiological agent responsible for cold-water vibriosis in salmonids as well as in gadidae^36^. Psychromonas bacteria are associated with sea ice environments^38^ and demonstrate a propensity to endure cold conditions within the cold chain. Weissella adds a potential probiotic dimension, though Acinetobacter and Rhodococcus bring ecological variability, with some strains displaying opportunistic pathogenicity. Tilapia demonstrates a microbiome featuring Acinetobacter, Pseudomonas, and Serratia. Lactococcus contributes to the potential probiotic aspect. Apart from Pseudomonas, caution is warranted due to the presence of potential pathogens, with Acinetobacter^32^ and Serratia. Tuna exhibits a microbiome including Bacteroides, Bifidobacterium, Chryseobacterium, Lactobacillus, and Parabacteroides. Bifidobacterium and Lactobacillus hint at probiotic potential. Chryseobacterium and Bacteroides imply a generally low pathogenic risk. Parabacteroides, commonly a gut commensal, adds a illustrational touch to the microbial landscape (Fig. 3).

**Figure 4:**
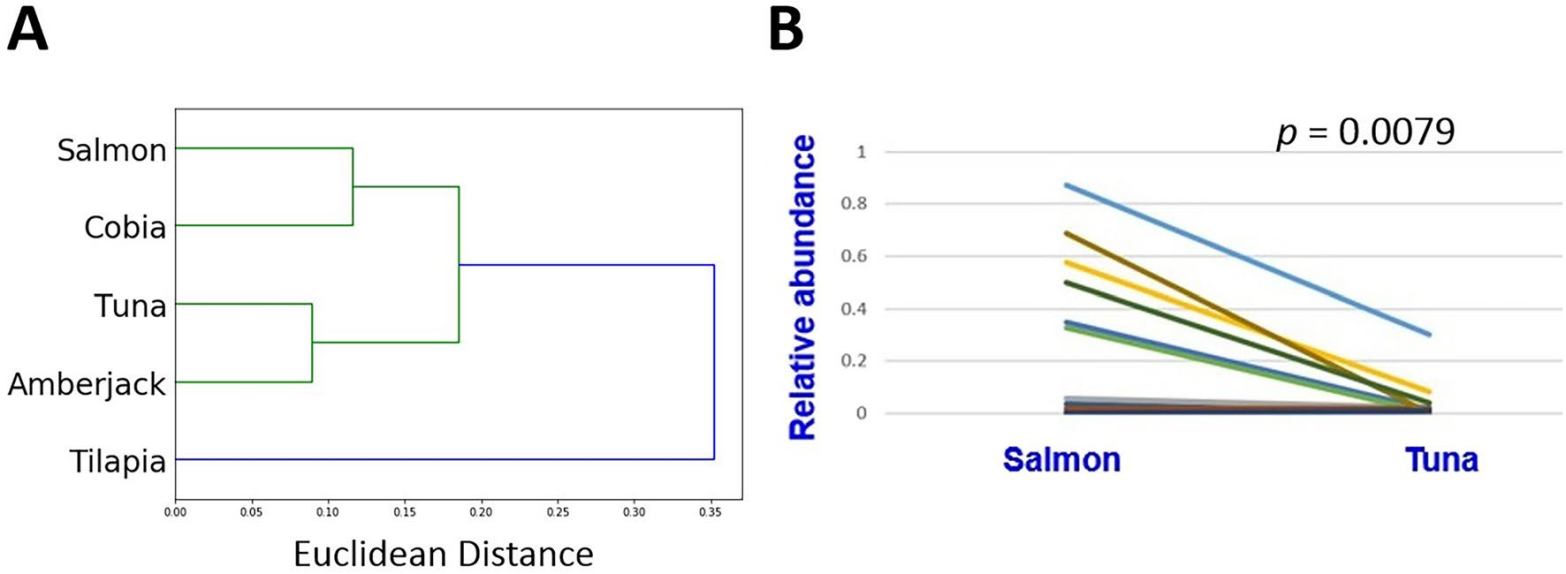
(**A**) The microbiome similarity tree constructed by the annotated microbiota, which demonstrates the highest similarity between amberjack and tuna, as well as cobia and salmon, with the tilapia distantly related to the other fish types. (**B**) The relative abundance of *Photobacterium* spp. DNA in salmon significantly exceeds that in tuna based on a paired comparison of all the restaurants that we investigated (Paired t-test; P = 0.0079). Each line represents a restaurant.

Using the 1232 OTUs successfully annotated to the genus level in this study, a similarity tree was constructed which unveiled a distinct affinity between amberjack and tuna, as well as cobia and salmon (Fig. 4A). The affinity represents shared commonality in their microbiome compositions. These four types are basically seawater fishes. Tilapia, which belongs to fresh water fishes, branch off separately and formed the most outskirt branch of the microbiome similarity tree (Fig. 4A) This is consistent with the difference between tilapia microbiome and other sashimi microbiome we observed in the NMDS plot (Fig. 2G) and the Shannon alpha diversity analysis (Fig. 2E), where salmon, cobia, tuna and amberjack have similar Shannon alpha diversity values, while tilapia’s values are toward the lower side. Since salmon and tuna represents two important branches of our microbiome tree, and are available from all the sample collection sites due to their gourmet popularity, we continue to perform paired statistical comparison, for evaluating differential microbiome abundance given the same restaurant. Among the 8 representative OTUs of salmon and 8 of tuna, we noticed that the relative abundance of *Photobacterium* DNA in salmon exceeds that in tuna in all the restaurants that we investigated (Fig. 4B). Paired t-test shows that the relative abundance of *Photobacterium* spp. is significantly higher in salmon than the other fish types (P = 0.0079).

## Discussion

The study explores the largely uncharted microbiome of sashimi at the time when it is served to customers to be consumed. Microbiome DNA remained in sashimi offer informative clues for us to reconstruct the microbiome. Deciphering the microbial composition of sashimi contributes toward our knowledge of food. The annotated microbiome profiles allowed us to generate a similarity tree (Fig. 4A). Interestingly, the tree generated by the microbiome seemed to reflect part of the evolution phylogeny. In the microbiome tree, amberjack and tuna are grouped together. They are both Perciformes, a diverse order that includes many families of bony fish. On the other hand, cobia (another Perciformes) and salmon (belonging to the Salmonidae family) are also grouped together. The tree is lastly joined with tilapia which belonging to the Cichlidae family (Fig. 4A). The resemblance of the microbiome tree and the host phylogenetic tree echoes the host-microbiome coevolution hypothesis^39^. The consistency of the two types of trajectory underscores the joint influence of both aquatic environments and host evolution on microbial composition.

This study demonstrated significantly higher proportions of *Pseudomonas* in tilapia sashimi compared to other fish sashimi (P = 0.0010). The Venn diagram illustrates a substantial number of unique OTUs identified only in one fish type, suggesting distinct microbial communities associated with salmon, tuna, amberjack, cobia, and tilapia, reaffirming the concept of host-specific microbial compositions. The alpha diversity analyses (the Chao1 index and the Shannon diversity index) demonstrate notable variations among fish types, with salmon exhibiting the highest richness. The Chao1 alpha diversity index showed that salmon sashimi displayed a greater degree of diversity in its microbiota in comparison to other fish species that we investigated (Fig. 2D). Salmon, belonging to the Salmonidae family and Salmoninae subfamily, is a popular food source worldwide. They travel between freshwater and saltwater habitats in their life cycles, and they are known to swim upstream the river to spawn. The high Chao1 alpha diversity is likely attributed to its distinctive life cycle, which contribute to the microbiota composition. Furthermore, the beta diversity analysis using Bray–Curtis dissimilarity index and NMDS plot reveal distinct clusters based on fish species, emphasizing the substantial impact of fish types on shaping the sashimi microbiome.

We found that *Photobacterium* is a signature genus of salmon, and the relative abundance of *Photobacterium* DNA in salmon significantly exceeds that in tuna based on a paired comparison in all the restaurants that we investigated (P = 0.0079; Fig. 4B). In this example, the microbiome composition of sashimi is significantly influenced by the type of fish species, regardless of the restaurants from which it was prepared and their supply chains, wherein human-related contamination might possibly arise. *Photobacterium* spp., which are gram-negative bacteria, belong to the *Vibrioceae* family. It is widely observed in the marine environment^40^. *Photobacterium* spp. has been reported to exist in salmon^41^ and Atlantic cod^42^ meat. It has also been reported in European plaice^43^. Photobacteriosis is the common bacterial disease affecting wild and farm cobia^44^. We discovered that *Photobacterium phosphoreum*, a bioluminescent bacterium living in symbiosis with marine organisms, is particularly enriched in salmon sashimi. This has been reported to be responsible of seafood spoilage and histamine fish poisoning, even in low temperature and anaerobic conditions^45,46^. The microbiome landscape not only deepen our understanding of sashimi microbiota but also offer valuable implications for food safety, quality assessment, and potential health considerations associated with probiotics. This understanding highlights the need for continued research into the complex interplay between diet, microbiota, and health, further underscoring the importance of mindful food choices in promoting overall well-being. That said, the knowledge is useful to customers only when the sashimi is correctly labeled. Our series of studies highlight the importance of correct labeling, enabling strategies for improving seafood safety and preventing seafood substitution.

## Conclusion

This study provided comprehensive information on seafood microbiome. Using DNA barcoding and deep sequencing, we profiled amberjack, cobia, salmon, tuna, and tilapia sashimi. Tilapia consistently showed elevated *Pseudomonas* levels (P = 0.0010). Our study underscores the intricate relationship between fish species, their environments, and their characteristic microbiomes. These findings not only validate prior observations but also shed light on species-specific microbial signatures, emphasizing the need for further exploration into the implications for both ecological and consumer health perspectives.

### Supplementary Information

Supplementary Tables.

## Data Availability

All data generated and analyzed during this study are included in this published article and its supplementary information files.
